# Seroprevalence of hepatitis A virus among Iranian soldiers 

**Published:** 2016

**Authors:** Morteza Izadi, Ali Aliakbar Esfahani, Hadi Hassannia, Nematollah Jonaidi Jafari, Fatemeh Rahmati Najarkolaei, Mohammad Saeid Rezaee-Zavareh

**Affiliations:** 1 *Health Research Center, Baqiyatallah University of Medical Sciences, Tehran, Iran*; 2 *Health Management Research Center, Baqiyatallah University of Medical Sciences, Tehran, Iran*; 3 *Department of Medical Immunology, Tehran University of Medical Sciences, Tehran, Iran*; 4 *Student’s Research Committee, Baqiatallah University of Medical Sciences, Tehran, Iran*; 5 *Middle East Liver Disease Center, Tehran, Iran *

**Keywords:** Hepatitis A virus, Prevalence, Seroepidemiological study, Military personnel

## Abstract

**Aim:**

This study aims to investigate the seroprevalence of HAV immunity among Iranian soldiers and determine whether vaccination should be given to military draftees.

**Background:**

Hepatitis A virus (HAV) is highly contagious in individuals living in crowded conditions such as military centers. To the best of our knowledge, there are limited data about HAV prevalence among Iranian soldiers.

**Patients and methods:**

In this cross-sectional study, a total of 1554 soldiers were recruited through a random clustering sampling. Serum anti-HAV antibody was measured by Enzyme-linked immunosorbent assay (ELISA). Statistical analysis was performed using SPSS.

**Results:**

A total of 1554 male soldiers with age ranged from 18 to 34 years (mean age: 21.2±1.9 years) at baseline were evaluated. Overall, 80.3% of the analyzed specimens were anti-HAV seropositive. Seroprevalence rates significantly increased with the age.

**Conclusion:**

Our results suggest that vaccination for HAV is not necessary for Iranian military draftees. However, the vaccination is recommended for high-risk groups, including anti-HAV seronegative soldiers.

## Introduction

 Hepatitis A is an acute type of liver disease caused by hepatitis A virus (HAV). HAV is an RNA virus classified as a picornavirus. It is transmitted mostly through the fecal-oral route, direct contact with an infectious person; as well as contaminated food or water (1). This virus is more prevalent in low socioeconomic, poor hygiene, and directly related to overcrowding regions where sanitary and hygienic conditions are not well maintained (2, 3). Approximately, 1.4 million new cases of hepatitis A infection annually occur worldwide. It is said that 11 to 22% of them need hospitalization (4).

Studies showed that the prevalence of anti HAV antibodies in the general population varies from 15% to nearly 100% in different parts of the world. Iran is located in an endemic region for HAV infection (5).

Most HAV infections are self-limited; however, severe symptoms and complications associated with acute hepatitis A increase with age. At present, it appears from the data, due to improvements in sanitation and hygiene, the age of infection by HAV has shifted from childhood to adolescence (6). When HAV infection occurs in adulthood, rate of jaundice and fulminant liver failure is much higher. Furthermore, it requires several days or weeks of hospitalization and causes absenteeism from work for several weeks. Thus, adulthood HAV infection can be costly in terms of direct medical costs and the absence of infected person from work (7). 

Immunization has been available and HAV infection could be prevented by vaccination, but is not yet widely used. Cost and feasibility are two major problems to implement HAV vaccination programs (1, 3).

To the best of our knowledge, there are limited data regarding HAV prevalence among Iranian soldiers. The aim of this study is to investigate current seroprevalence of HAV immunity among Iranian soldiers to determine whether vaccination should be given to military draftees.

## Patients and Methods

**Study Design and Population**

In this cross-sectional study, 1554 soldiers (all men) were selected among the military draftees through a random cluster sampling from 2011 to 2013 (Tehran, Iran). Basic clinical and demographic information and common risk factors such as family history of HAV, hometown of soldiers, source of water supply, and method of sewage disposal were collected using a questionnaire, and informed consent was obtained from all participants. 

**Figure 1:**
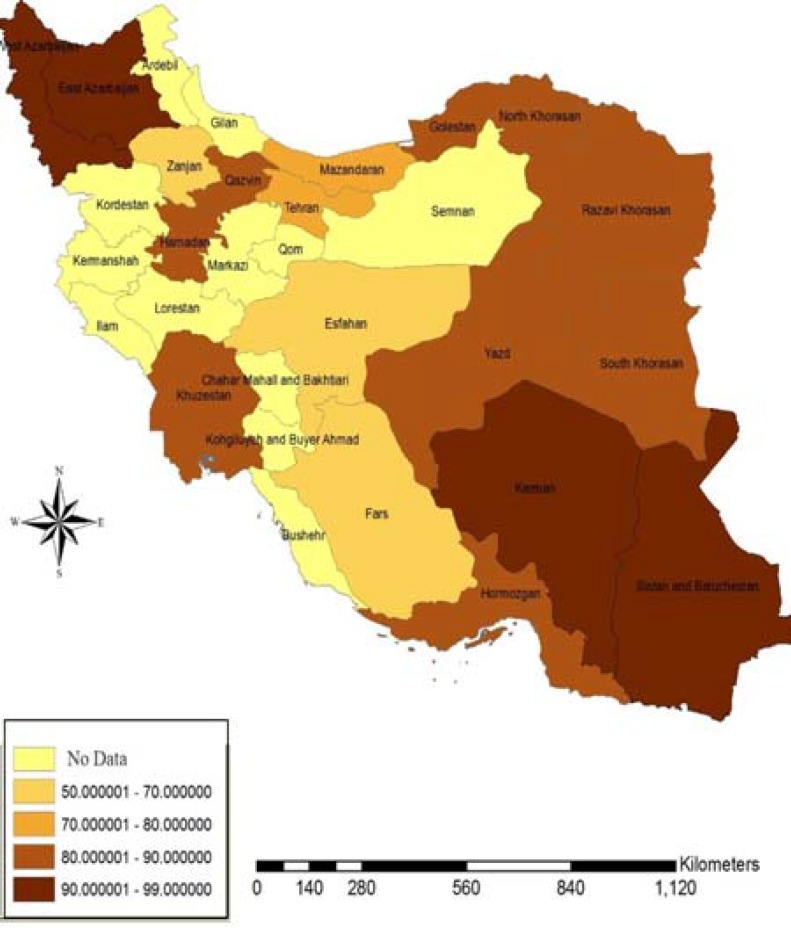
The GIS Map of HAV Seroprevalence Rates in 15 Provinces of Iran (Based on the Gathered Data of Soldiers of Different Provinces of Iran)

**HAV Identification**

Our method for determining HAV infected patients was similar to Ghorbani, et al. (8). To determine anti-HAV, 5 ml of peripheral blood was taken from entire Participants. Serum separated by centrifugation at 5,000 rpm for five minutes and stored at -20°C until use. The presence of anti HAV antibodies was measured using a qualitative ELISA method (Abbott- HAV-Ab, AB META-AXSYM, Germany) according to the manufacturer’s instructions. The results were reported as positive or negative. The positive cases of anti-HAV were considered as an immune to HAV through either vaccination or natural infection. On the other hand, negative cases for anti-HAV antibodies should be considered susceptible to HAV infection.

**Data Analysis**

Data were analyzed using SPSS version 17 (SPSS Inc., Chicago, IL, USA). Results were compared between seropositive and seronegative groups using T test and chi square. Statistical significance was accepted at a level of P<0.05. ArcMap 9.3 GIS software (ESRI, Redlands, CA, USA) was used to produce the map (Figure 1). 

## Results

During the entire 15-month study period, 1554 soldiers (all men) included in this study. The mean age of participants was 21.2±1.9 (ranged from 18 to 36) years. From 1554 soldiers, 1248 (80.3%) had anti-hepatitis A virus antibody, while the other 306 (19.7%) were seronegative for HAV. For evaluating the effect of age on seroprevalence, participants were categorized into three age groups; group A: < 20 years, group B: 20–30 years, and group C: > 30 years. Statistically, the seroprevalence rate increased significantly with age: from 72.2% in age group A to 79.1% in group B, and to 92.4 % in Group C (P= 0.01) (Table 1). 

**Table 1:** Age-specific seroprevalence of Anti HAV antibody in subjects

Age group	Negative	Positive
< 20	117 (27.8%)	304 (72.2%)
20–30	162 (20.9%)	616 (79.1%)
> 30	27 (7.6%)	328 (92.4% )
Total	306 (19.7%)	1248 (80.3%)

Of our seropositive subjects, 52.03% were from rural area and 47.96% were from urban regions. There was no significant difference between these groups (P= 0.6). Other variables, including educational level, water supply, as well as wastewater and sewage disposal systems did not influence HAV seroprevalence rate. A prevalence report based on the province of soldiers can be seen in the figure 1. 

## Discussion

The Middle East area is endemic for HAV infection. Most countries in this area have more than 90% HAV seropositivity rate (9, 10). Previous reports based on healthy blood donors in Iran, report a rate of 95% or even more in adults (5, 11). The prevalence of HAV has been inversely influenced by the level of sanitation, personal hygiene and socioeconomic standing, and is directly related to overcrowding. Vaccination is the best prevention method for high-risk groups like military soldiers (12). 

In this cross-sectional study, we investigated the seroprevalence of HAV among Iranian soldiers. We found an overall seroprevalence of 80.3% in our population, which tallies with results of some similar studies (12-14). However, compared to previous studies less seroprevalence was observed, which may have been caused by improvements in food and water hygiene especially in military centers in Iran (15).

HAV infection can be more severe when it occurs in adulthood. In our analysis, we showed a significant correlation between age of the participants and HAV seroprevalence. Other available literatures have also suggested this gradual shift (16, 17). Important reasons for this shifting can be quality of water sources and improvement in the socio-economic status (18). However, we could not find a significant effect of water supply and educational level on the HAV seroprevalence. It may be due to that all our cases are from military centers.

Ghorbani, et al. reported in their study that 97.63% of army draftees were immune to hepatitis A virus and indicated that vaccination for HAV is not necessary for Iranian military conscripts (8). Also, the hyper endemic pattern was confirmed by comparison with data reported by earlier published investigations in different restricts of Iran (19). Our results showed a high prevalence rate of infection in some provinces like Ahwaz, Hamedan, Tehran, Hormozgan, Golestan, Qazvin, Shiraz, Azarbaijan and Yazd, while HAV seroprevalence in Esfahan, Zanjan and Fars provinces demonstrated a lower rate of infection (Table2, Fig 1). These results may implicate an epidemiological transition to lower rates of infection in particular parts of the country.

**Table 2:** Anti HAV antibody seroprevalence rate by sites of residence from different provinces of Iran

Sites of residence	Positive	Sites of residence	Positive
Azarbaijan	58 (92.04%)	Zanjan	23 (58.09%)
Gazvin	97 (83.64%)	Isfahan	237 (51.21%)
Golestan	13 (89.14%)	Fars	141(52.13%)
Hamadan	18 (86.29%)	Tehran	327 (79.36%)
Hormozgan	9 (87.73%)	Sistan Baluchestan	16 (95.48%)
Mazandaran	83 (73.57%)	Kerman	23 (97.31%)
Khorasan	93 (86.92%)	Yazd	49 (89.48%)
Khozestan	61 (80.65%)		

During the past two decades, many countries in Asia enhanced in socioeconomic status associated with urbanization, health education, lifestyle, as well as access to improved food and water hygiene (20-22). This pattern may reduce the population’s immunity, therefore more people remained susceptible to HAV infection.

Our main limitation was related to the type of study that was a cross-sectional one. We could not get samples from all soldiers of military centers in Tehran. Therefore, we had to use a random cluster sampling. Selected soldiers were also at the first month of their military service. Based on these issues, we tried to estimate HAV prevalence of each province of Iran based on the hometown of soldiers.

In conclusion, according to our findings and literature reviews, it seems that the immunity of HAV in soldiers is lower than the general population. However, the seroprevalence is still very high for recommending routine vaccination in general military soldiers. On the other hand, there are evidences that show HAV vaccination among susceptible cases in countries of the Middle East and North Africa. It is said that this can lead to reduce mortality and morbidity (23).

Therefore, we recommend monitoring of HAV seroprevalence in the general military soldiers to determine high-risk groups, including anti-HAV seronegative soldiers for HAV vaccination.

## Note

**(**Please cite as:** Izadi** **M, Aliakbar Esfahani** **A, Hassannia** **H,** **Jonaidi Jafari** **N, Rahmati Najarkolaei** **F, Rezaee-Zavareh MS. Seroprevalence of hepatitis A virus among Iranian soldiers. Gastroenterol Hepatol ****Bed Bench**** 2016;9(2):100-104).**
